# Auditory processing and its cognitive correlates in older adults with mild cognitive impairment

**DOI:** 10.1186/s12877-025-05997-4

**Published:** 2025-05-24

**Authors:** Leelavathi Thamizhmani, Kanaka Ganapathy, Hari Prakash Palaniswamy, Arivudai Nambi Pitchaimuthu, Prabha Adhikari M. R.

**Affiliations:** 1 Department of Speech and Hearing, Manipal College of Health Professions, Manipal Academy of Higher Education, Madhav Nagar, Manipal, 576104 India; 2 Center for Hearing Science, Department of Audiology, All India Institute of Speech and Hearing, Naimisham Campus, Manasagangothri, Mysuru, Karnataka 570006 India; 3 Department of Geriatric Medicine, Yenepoya Medical College, Yenepoya (Deemed to be University), Deralakatte, Mangaluru, Karnataka 575018 India

**Keywords:** Auditory processing, Mild cognitive impairment, Cognition, Factor analysis, Clinical utility, Left ear extinction, Temporal processing

## Abstract

**Introduction:**

Age-related central auditory processing disorder (CAPD) is linked to cognitive decline in older adults, potentially preceding Mild Cognitive Impairment (MCI) by several years. While studies indicate that all auditory processing domains are impacted, it remains unclear which domain most significantly correlates with cognitive functions in MCI, warranting further investigation into these relationships. The current study investigated auditory processing deficits and their relationship with cognitive performance in older adults with MCI.

**Method:**

The study recruited 70 participants aged 60 to 72 years, divided into two groups: MCI(*n* = 35) and healthy controls (*n* = 35) based on Montreal Cognitive Assessment scores. Comprehensive central auditory processing and cognitive assessments were done.

**Results:**

The MCI group showed significant deficits across all auditory processing and cognitive domains. The enhanced right ear advantage in the dichotic test could be due to corpus callosum atrophy affecting left ear processing. Different factor structures in MCI suggest that they relied more on attentional resources for complex auditory tasks. Temporal processing tests showed high sensitivity in identifying MCI, with strong AUC and R² values, underscoring their clinical relevance.

**Conclusion:**

Temporal processing deficits could serve as an early screening tool for cognitive decline in older adults. Larger studies targeting individuals with age-related hearing loss (ARHL) and MCI are essential, given the prevalence of ARHL in this population. Research should also examine the impact of tailored auditory training on cognitive function in MCI to inform interventions.

**Trial registration:**

The study was registered in the Clinical Trials Registry of India (CTRI/2023/06/054277) on 21/06/2023 (http://ctri.nic.in/).

**Supplementary Information:**

The online version contains supplementary material available at 10.1186/s12877-025-05997-4.

## Background

Hearing impairment is highly prevalent among older adults [1]. This age-related auditory decline involves peripheral and central hearing changes and is termed peripheral and central presbycusis, respectively [2–5]. Central Auditory Processing Disorder (CAPD) is characterized by difficulty understanding speech in noisy environments, with competing speech, or when speech is distorted despite having a normal peripheral hearing. Older adults often experience difficulties understanding speech when there is background noise. While their peripheral hearing sensitivity plays a role in speech recognition, the ability to recognize speech heavily relies on central auditory processing skills, especially temporal resolution.

Temporal processing is the brain’s ability to interpret sounds over time, which is essential for understanding complex auditory information, including speech. It underlies various auditory processing skills such as pitch discrimination, intonation recognition, and linguistic information extraction—all crucial for speech comprehension [6, 7]. While the primary auditory cortex and temporal lobe primarily handle these functions [8], other regions like the parietal and occipital lobes also play a role [9]. These areas are interconnected within key cognitive networks, including the frontotemporal network for language processing and the dorsal attention network for auditory working memory. Notably, these regions are vulnerable to early neurodegenerative changes in Mild Cognitive Impairment (MCI) [10].

Several studies have shown that temporal processing declines with age, and individuals with MCI exhibit significantly poorer performance on temporal resolution tasks compared to age-matched controls [11, 12]. In older adults with CAPD, increased cognitive resources are allocated for auditory comprehension [13, 14], and fewer resources are available for other cognitive activities, such as working memory [15]. This potentially accelerates cognitive decline [16]. Impairments in temporal processing tests, such as Gaps-in-Noise (GIN) have been linked to early cognitive decline and may serve as potential markers for identifying cognitive decline [17]. Furthermore, neurophysiological studies have shown that MCI is associated with altered cortical responses to temporally complex auditory stimuli, as evidenced by reductions in mismatch negativity (MMN) and cortical auditory evoked potentials (CAEPs) [18, 19].

While temporal processing is crucial, other auditory processing skills also play significant roles in understanding speech like binaural integration and speech in noise [20]. Nevertheless, the comprehensive assessment of central auditory processing in MCI is limited. Commonly used assessments include the Low-redundant tests, including Synthetic Sentence Identification- Ipsilateral Competing Message (SSI- ICM) [21, 22], Speech Perception in Noise (SPIN) [23] and dichotic tests such as Dichotic Digit tests (DDT) [24–26], and Dichotic Sentence Identification test (DSI) [21, 22]. A recent meta-analysis [27] suggests that individuals with MCI performed poorly in all CAPD tests compared to cognitively healthy controls. While the meta-analysis did not explicitly discuss peripheral hearing loss, a review of the included studies indicates that most required participants to have pure-tone thresholds below 40 dB HL, thereby minimizing the influence of significant peripheral hearing loss on auditory processing outcomes.

Despite the growing evidence linking auditory processing to cognitive decline in MCI, significant gaps remain in our understanding. Existing research has predominantly focused on identifying group-level deficits and establishing a correlation between auditory processing and cognitive decline. However, the extent of this relationship is unclear. There is a lack of understanding of the underlying constructs of auditory processing deficits and cognition in MCI. This study aimed to (1) determine whether individuals with MCI exhibit significant deficits in auditory processing compared to cognitively healthy older adults, (2) examine whether the underlying constructs and relationships between auditory processing abilities and cognitive function differ between MCI and control groups, and (3) identify which specific auditory processing deficits have the strongest predictive or linear relationship with cognition in MCI.

Understanding the relationship between auditory processing deficits and cognitive decline is essential for enhancing clinical and rehabilitation approaches in older adults. Identifying specific auditory processing impairments associated with MCI can aid in the development of early screening protocols, allowing for the detection of cognitive decline at a stage where interventions may be more effective. Additionally, this knowledge informs the design of targeted auditory training programs, which have shown promise in improving both speech processing and cognitive outcomes in older adults [28, 29].

## Method

### Overview

The current study was approved by the Institutional Research Committee and the Ethics Committee at Kasturba Hospital, Manipal (IEC1: 352/2022). Participants have given written informed consent before initiating the study. The study involved older adults between 60 and 72 years of age with a minimum of 10 years of education. All the participants were recruited from communities in the districts of South Canara, Karnataka, India. Cognitive screening was performed using the Montreal Cognitive Assessment (MoCA) [30] in Kannada, which was culturally adapted and validated and is available from https://mocacognition.com/.

In addition, hearing screening was also done. The participants whose hearing threshold were ≥ 40 dBHL at octave frequencies (250 Hz, 500 Hz, 1 kHz, 2 kHz, 4 kHz, and 8 kHz) were excluded from the study. The inclusion of individuals with hearing thresholds of ≤ 40 dB HL aimed to reduce the confounding impact of peripheral hearing loss on central auditory processing (CAP) performance. All auditory processing tests were conducted at suprathreshold levels or at the most comfortable level (MCL) for each participant, ensuring that even those with mild hearing loss could perform the tasks accurately. Setting the cutoff at 40 dB HL was practical for participant recruitment and minimized interference with central auditory processing outcomes, especially given the high prevalence of age-related hearing loss in older adults. Additionally, participants with a history or presence of neurological disorders such as stroke, epilepsy, brain injury, or tumours, as indicated by medical records and psychological illness based on the Patient Health Questionnaire-9 – Kannada (PHQ-9) [31, 32], were excluded.

The participants were categorized into two groups based on their MoCA scores: those with scores ranging from 19 to 25 were classified into the MCI group, whereas those with scores above 25 were classified into the control group. Participants with MoCA scores less than 19 and those who were undergoing cognitive or behavioural interventions such as yoga, physical exercise, meditation, or mindfulness were excluded from the study.

Participants and their caretakers were informed of their MoCA scores and the implications of MCI. The study procedures were explained in detail, and each participant received an information sheet approved by the ethics committee. They had sufficient time to review the information before providing written informed consent, which was also witnessed and signed by their caretakers.

### Test procedure

All consented participants underwent a pure tone audiometry test using a GSI 18 portable audiometer (ANSI S3.6 Audiometer, type 4) coupled with TDH39 headphones (Grason-Stadler INC, Minnesota, US). Individuals with hearing thresholds ≤ 40 dBHL at 250 Hz, 500 Hz, 1 kHz, and 2 kHz were included in the study. A comprehensive central auditory processing test was subsequently administered, which included the speech-in-noise test in Kannada (QuickSIN-K), the dichotic digit test (DDT) in Kannada, and temporal processing tests, such as the temporal fine structure‒adaptive frequency (TFS‒AF), frequency modulation difference test (FMDL), adaptive threshold of temporal resolution (ATTR), and modulation detection threshold (MDT). All the tests were administered via a Windows 10 HP Pavilion laptop connected to calibrated Audio-Technica ATH-M20x headphones.

Following the audiological tests, participants’ cognitive performance was evaluated via behavioural tests, such as the Attentional Network Test (ANT), Mental Rotation Test (MRT), Digit Span Test- forward & backward and Trail Making Test- Part A and B (TMT). All the behavioural tests were developed in E-prime software (version 3.0). All the participants were comfortably seated in front of a laptop for test administration, and the mouse and keyboard were used as response buttons for the respective tests.

### Central auditory processing tests

#### QuickSIN-K

Standardized sentences in Kannada [33] were used in this study to measure participants’ ability to understand speech in the presence of background noise. The participants were presented with a list of seven Kannada sentences, each containing five keywords played in speech babble at different signal‒to‒noise ratios (SNRs). The SNR progressively decreased by 3 dB with each sentence, ranging from 8 dB to -10 dB. Each participant was asked to repeat as many words as possible from each sentence. The sentences were played at the most comfortable level (MCL) for each participant. A score of one was given for each correctly repeated keyword, with a total of 5 points possible per sentence. The SNR loss was calculated using the formula [SNR loss = 22.5 - total score]. It took approximately 10 min to complete this test.

#### DDT

DDT in Kannada assessed binaural integration skills (Bhat et al., 2020). Different Kannada numbers were presented to each ear at the same time. The stimulus pairs are double digits (two numbers for each ear) presented at 70 dB SPL. It was performed under three conditions: free recall, forced right, and forced left. In free recall, the patient repeated all the digits heard, regardless of ear or sequence. A score of 1 was given as a Double Correct Score (DCS) for the correct recall of all four digits. If a person correctly recalls the stimuli from the left ear while one or no stimuli that are presented in the right ear, a score of 1 is given as the left correct score (Total L). Conversely, the right correct score (Total R) is calculated under the same conditions for correct responses from the right ear. In the forced condition, the participants were asked to repeat the digits heard either in the right ear or in the left ear, respectively. Forced right correct scores (Forced RCS) and forced left correct scores (Forced LCS) were calculated separately. A score of 1 was assigned when all stimuli from the attended ear were recalled correctly. The right ear advantage (REA) was determined by subtracting the left ear score from the right ear score (Total R – Total L). It took approximately 30 min to complete this test.

#### Temporal processing tests

The temporal processing tests were carried out using a custom script developed in an adaptive manner using MATLAB (R2021a Update 8) (The MathWorks, Inc., Natick, MA, US).

##### TFS- AF

Inter-aural phase discrimination task by ​Fullgrabe et al. (2017)​ was adopted in this study. The test assesses the participant’s ability to detect changes in Interaural Phase Difference (IPD) using two intervals and an alternative forced choice method. Two consecutive intervals were created for each trial with a 500 msec interstimulus interval. Each interval contained four consecutive 400 msec tones separated by 100 msec. In the standard interval, the IPD of all four tones was 0°. In the target interval, the first and third tones were the same as in the standard, whereas the second and fourth tones differed in their IPDs by 180°. The IPDs were constant, and the frequencies were adaptively adjusted using a two-down, one-up adaptive procedure. The starting frequency was 200 Hz and increased up to 4000 Hz. The stimulus is presented at 30 dBSL in each ear. The patient’s task was to indicate which of the intervals contained the sequence of tones that appeared to be moving (differed in IPD). The step size for frequency adjustments was 1.25, with the frequency increasing after two consecutive correct responses and decreasing after one incorrect response. A total of eight reversals were recorded, and the last six reversals were used to calculate the threshold. The threshold in Hz was determined as the highest frequency at which participants could accurately identify the IPD. It took approximately 15 to 20 min to complete this test.

##### FMDL

This test measures the ability to detect the smallest change in modulation frequency by using three intervals and an alternative forced choice method. In the test, three tones were played in succession: one was modulated, and the other two were unmodulated. The target stimulus is a frequency-modulated signal sampled at 44.1 kHz, lasting 1250 milliseconds. The carrier frequency was set to 500 Hz, and a fixed modulation rate of 2 Hz was used with an initial modulation depth of 25. An adaptive procedure with a two-down, one-up algorithm was employed to determine the participant’s threshold. The stimuli were presented at 70 dB SPL. The modulation depth was adjusted based on the participant’s responses, with a step size of 0.5. A total of eight reversals were recorded during the procedure, and the last six reversals were used to calculate the threshold. The threshold is the modulation depth (Hz) at which participants were able to detect the smallest change in modulation frequency. Participants were asked to identify the interval containing the frequency-modulated tone. It took approximately 20 min to complete this test.

##### ATTR

This test measures the smallest temporal gap in an auditory signal that a listener can detect. The test had three intervals, all of which were broadband noise, with only one interval containing gaps of varying durations. In this study, the initial gap duration was set to 10 msec. The stimuli were presented at 70 dB SPL. An adaptive two-down, one-up staircase procedure with step size of 0.5 was employed to adjust the gap duration based on the participant’s responses. Correct detections of the gap led to a decrease in the gap duration by a factor of 0.5, while incorrect responses increased the gap duration by the same factor. This staircase method continued until eight reversals were achieved, and the threshold (msec) was calculated as the geometric mean of the last six reversals. It took approximately 15 min to complete this test.

##### MDT

This test measures the smallest detectable modulation depth at a fixed modulation frequency. The stimuli were broadband noise signals sampled at 44.1 kHz, each lasting 500 milliseconds. The noise was amplitude-modulated at a fixed frequency of 8 Hz. The initial modulation depth was set to 0.5, and the threshold was determined using an adaptive procedure that aimed to achieve four reversals with a step size of 1.25. During the experiment, participants were presented with noise intervals and were required to identify the interval containing the modulated noise. The modulation depth was adjusted iteratively until the required number of reversals was obtained. The stimuli were presented at 70 dB SPL. The threshold modulation depth (dB) was calculated as the geometric mean of the modulation depths at the last two reversals and then converted to decibels (dB). It took approximately 20 min to complete this test.

### Cognitive assessment tests

#### ANT

This study employed a modified version of the ANT adapted from Fan et al. (2002) [34]. It is a cognitive task designed to assess three distinct components of attention: alerting, orienting, and executive control. Alerting reflects the ability to maintain a state of readiness, orienting involves selecting relevant sensory information, and executive control refers to the ability to resolve conflict among competing responses. The stimuli consisted of a series of arrows pointing either rightwards or leftwards. The central target was an arrow pointing either left or right, flanked by two arrows on each side. These flankers could point in the same direction as the target (congruent condition), the opposite direction (incongruent condition), or be a nondirectional symbol such as a diamond (neutral condition) (refer to supplementary material for three flanker conditions).

The experiment comprised a single block of 120 trials, presented randomly. It included three cue conditions, two stimulus positions, two stimulus directions and three congruency conditions. Each trial consisted of five components: an initial fixation for 1000 milliseconds, followed by a cue displayed for 1000 milliseconds, and then a middle fixation period of 1000 milliseconds. After this, the stimulus, which included a target and flankers, was shown for a maximum of 2000 milliseconds or till the participant’s response. The task required participants to quickly indicate the direction of the central arrow by clicking the right or left mouse button. All participants initially completed a practice block consisting of 12 trials, during which feedback was provided on their response accuracy and reaction time.

There were three warning conditions for measuring alerting and orienting attention: no cue, centre cue, and spatial cue. For the no-cue condition, there was only fixation, followed by the target and the flanker. For the centre cue, an asterisk at the fixation location was shown for 1000 milliseconds, involving alerting. For the spatial cue, the asterisk was at the target position, with the same timing as the center-cue and no-cue trials. The spatial cues were always valid, meaning that they were displayed at the target locations. Both alerting and orienting were expected under these conditions. It took approximately 30 min to complete this test.

The alerting effect was calculated by subtracting the mean reaction time (RT) (msec) of the centre cue conditions from the mean RT of the no-cue conditions (RT_no cue_ – RT_centre cue_). The orienting effect was calculated by subtracting the mean RT of the spatial cue conditions from the mean RT of the centre cue (RT_center cue_ – RT_spatial cue_). Finally, conflict or executive control was calculated by subtracting the mean RT of all congruent flanking conditions, summed across cue types, from the mean RT of incongruent flanking conditions (RT_incongruent_ – RT_congruent_).

#### MRT

The mental rotation test was designed to assess spatial visualization ability. The stimuli consisted of six alphabetic letters (F, G, L, P, R), each shown in four different conditions: standard, mirrored, and both forms rotated clockwise or counterclockwise at a random angle. The test comprised 100 trials (5 letters, 4 conditions, 20 samples presented in 5 cycles), with each of the letters and conditions presented randomly. Each trial began with a fixation cross (+) displayed for 1000 milliseconds, followed by the stimulus, which was shown for up to 5000 milliseconds or until the participant responded. The participants were instructed to identify whether the presented letter was the standard letter or a mirrored version (regardless of rotation). They were instructed to press ‘1’ for standard letters and their rotation and ‘2’ for mirrored letters and their rotation. Prior to the main test, the participants completed a practice block of 10 trials using only the letter P, during which they received feedback on their responses. Reaction times (msec) and response accuracy (percentage) were recorded by the software for each trial. It took approximately 30 min to complete this test.

#### Digit span test- forward and backwards

This test was used to assess short-term memory and working memory [35]. In this test, participants were presented with a series of digits on a screen. The sequences ranged from a minimum length of 2 digits (e.g., 68) to a maximum length of 8 digits. Each trial commenced with a 1000-millisecond fixation period, followed by a 1000-millisecond stimulus presentation. A command prompt subsequently appeared, accompanied by a text box in which the participants typed their responses. The participants were instructed to remember and type the digits in the exact order in the forward test and in reverse order in the backwards test. For each sequence, if the participant successfully recalled the digits in the correct order, a longer sequence was presented. In the case of an incorrect response, a second sequence of the same length was administered. This adaptive procedure continued until the participant failed to recall a sequence correctly or reached the maximum length of eight digits. The longest sequence that was recalled correctly, and the participants’ reaction times (msec) were recorded. The backwards test followed the same procedure, with participants being required to recall the digits in the reverse order of their presentation. It took approximately 30 min to complete this test.

#### TMT

This test assesses processing speed [36]. There were two parts to this test. In TMT-A, participants were instructed to connect 25 encircled numbers sequentially (1, 2, 3, etc.), which were randomly distributed on the computer screen. In TMT-B, participants are required to alternate between numbers and Kannada letters, which are also randomly distributed on the screen (1, , 2, , etc.). Participants used a computer mouse to click and connect the targets in the correct sequence. They were instructed to complete the task as quickly and accurately as possible. The researcher demonstrated a sample of each task before the actual test to ensure comprehension. The time taken (msec) to complete each test part was recorded in seconds. It took approximately 20 min to complete this test.

### Statistical analysis

All data were analyzed using Jamovi software (version 2.4.8). Continuous variables are presented as the median and interquartile range (IQR). To compare the group differences among all administered cognitive and auditory processing tests, the Mann-Whitney U test was used. To explore the predictive linear relationship between auditory processing abilities and cognitive function in individuals with MCI, multiple regression analysis was conducted. In addition, ROC analysis was performed to assess the sensitivity and specificity of auditory processing tests in distinguishing older adults with and without MCI. Additionally, exploratory factor analysis (EFA) was conducted to examine the underlying structure of cognitive and auditory measures in both groups, which may provide further insights into shared underlying constructs influencing performance across domains. The Kaiser-Meyer-Olkin (KMO) measure was used to assess sampling adequacy, and Bartlett’s sphericity test ensured that the data met the assumptions for factor analysis.

## Results

### Participant characteristics

For this study, the sample size was calculated using g power software. At a 5% level of significance, 90% power and an effect size of 0.8, the minimum sample size required to compare the means of two groups according to the formula is *N* = 56 (i.e., 28 per group). Incorporating 25% dropout rate, the sample size required was 70 − 35 per group.

All the participants were recruited by conducting hearing and cognitive screening camps in communities of South Canara, Karnataka, India. A total of 216 individuals participated in cognitive and hearing screenings. Of these, 53 were excluded for having hearing thresholds exceeding 40 dBHL. This left 163 participants, of whom 45 had MoCA scores ranging from 19 to 25, while 118 scored above 25. Of these, only 35 older adults with MCI and 40 older adults without MCI consented to this study. The test procedures took 3 to 4 h to complete; hence, the testing was conducted over two visits to reduce fatigue effects. Visit 1 included central auditory processing assessments, while Visit 2 focused on cognitive assessments. Within each session, tests were administered in a fixed order to ensure standardization across participants. Short rest breaks were provided between individual tasks, allowing participants to rest and maintain optimal cognitive engagement. This approach controlled for potential fatigue-related performance variations and standardised the testing protocol across all participants. Owing to a lack of motivation and various personal circumstances, 5 participants could not complete the entire study procedure. Therefore, the final sample consisted of 70 older adults. Among them, 35 participants were categorized as having MCI based on MoCA scores between 19 and 25, whereas the remaining 35 participants served as control subjects without cognitive impairment, with MoCA scores greater than 25.

The median age of participants was 65 years in the MCI group and 64 years in the control group, with no significant difference between the groups (*p* = 0.215). All participants had a minimum of 10 years of education, with no significant difference between them (*p* = 0.397). There were no significant gender differences between the groups (*p* = 0.904). The prevalence of diabetes was 40% in the MCI group and 54.3% in the control group. Hypertension was reported in 42.9% of MCI participants and 45.7% of controls. There were no statistically significant group differences in prevalence for either condition (p-values > 0.05). Duration of diabetes and hypertension were analyzed only for participants who reported having the condition and are summarized in Table 1.

**Table 1:** Summary of participant characteristics

Demographics	Group	*N*	Median (IQR)	Statistic	*p* value
**Age (years)**	MCI	35	65 (5)	507	0.215
	Control	35	64 (5)		
**Education (years)**	MCI	35	15 (5)	546	0.397
	Control	35	15 (3)		
**Diabetes (years)**	MCI	14	8.50 (9.25)	580	0.684
	Control	19	5 (7)		
**Hypertension (years)**	MCI	15	5 (6)	606	0.933
	Control	16	5 (5.5)		

### Pure-tone audiometry

The median hearing thresholds (dBHL) for both groups did not exceed 40 dBHL across frequencies ranging from 250 Hz to 8 kHz. There was no significant difference in the pure tone average (PTA) for frequencies of 0.5, 1, and 2 kHz or at speech frequencies (0.5, 1, 2, and 4 kHz) between both ears across the groups (Table 2).

**Table 2:** Pure tone average (dB HL) for both ears

PTA (dBHL)	Group	Median (IQR)	Statistic	*p* value
PTA Right	MCI	28.3 (10.8)	525	0.303
	Control	26.7 (6.67)		
PTA Left	MCI	31.7 (10)	523	0.293
	Control	26.7 (8.33)		
PTA speech Right	MCI	30 (10.6)	514	0.247
	Control	27.5 (6.25)		
PTA speech Left	MCI	32.5 (9.38)	519	0.270
	Control	28.8 (8.13)		

### Central auditory processing tests

The Mann‒Whitney U test was conducted to compare the mean difference between the two groups for all the auditory processing tests. Holm’s sequential correction method was used to adjust for multiple comparisons within the central auditory and cognitive domains separately, ensuring control of the family-wise error rate. The MCI group performed significantly poorer than the healthy controls in DDT. Total right ear scores were not statistically significant, but the MCI group demonstrated significantly better right ear advantage than controls. Other CAPD tests, like the temporal processing tests (TFS-AF, FMDL, TMTF, & ATTR), were also significantly different between the two groups. (Table 3) (refer to supplementary material for box plots). After Holm correction, the following central auditory measures remained statistically significant (adjusted *p* < 0.05): DCS, Total L, Forced LCS, REA, ATTR, MDT, TFS-AF, Forced RCS, and FMDL. QuickSIN did not remain significant (adjusted *p* = 0.064), and Total R was non-significant (adjusted *p* = 0.253).

**Table 3:** Comparison of central auditory processing between MCI and healthy controls

Dichotic Digit Test (DDT)					
Tests	Group	Median (IQR)	Statistic	*p*- value	Holm-adjusted *p*-value
DCS	MCI	2 (3)	211	< 0.001**	0.011*
	Control	10 (4)			
Total R	MCI	12 (6)	517	0.253	0.253
	Control	11 (2)			
Total L	MCI	4 (3)	202	< 0.001**	0.011*
	Control	12 (2.75)			
Forced RCS	MCI	10 (6)	368	0.002*	0.008*
	Control	13 (0.75)			
Forced LCS	MCI	7 (6)	211	< 0.001**	0.011*
	Control	13 (0)			
REA	MCI	6 (5)	220	< 0.001**	0.011*
	Control	0 (1.75)			
**Speech in Noise**					
QuickSIN-K (SNR in dB)	MCI	5.50 (2)	439	0.032*	0.064
	Control	5.50 (1)			
**Temporal Processing Tests**					
FMDL (Hz)	MCI	6.84 (8.05)	372	0.005*	0.015*
	Control	3.65 (3.83)			
MDT (dB)	MCI	-6.94 (3.01)	239	< 0.001**	0.011*
	Control	-9.82 (1.94)			
ATTR (msec)	MCI	13 (6.64)	302	< 0.001**	0.011*
	Control	4.9 (5.38)			
TFS- AF (Hz)	MCI	225 (5.6)	190	< 0.001**	0.011*
	Control	366 (214)			

**p < 0.001, *p < 0.05revealed significant differences between the two groups.

Total R- Right ear scores; Total L- Left ear scores; Forced RCS- Right correct scores in forced condition; LCS - Left correct scores in forced condition

### Cognitive tests

The cognitive performance of individuals in the MCI group was notably poorer than that of those in the control group across all the administered tests, as shown in Table 4. The Mann‒Whitney U test revealed significant differences between the two groups. All the variables showed statistically significant differences between the groups even after Holm adjustment (all adjusted *p* = 0.011).

**Table 4:** Comparison of cognition between MCI and healthy controls

Cognitive Tests	Group	Median (IQR)	Statistic	*p*- value	Holm-adjusted *p*-value
DS- forward RT (msec)	MCI	6490 (640)	121	< 0.001**	0.011*
	Control	4935 (559)			
DS- backward RT (msec)	MCI	7686 (3215)	132	< 0.001**	0.011*
	Control	4221 (783)			
DS- forward length	MCI	4 (1)	160	< 0.001**	0.011*
	Control	6 (1)			
DS- backward length	MCI	3.33 (1)	139	< 0.001**	0.011*
	Control	5 (1)			
TMT- RT number (msec)	MCI	92,672 (14553)	134	< 0.001**	0.011*
	Control	70,018 (8556)			
TMT- RT mixed (msec)	MCI	305,414 (67792)	150	< 0.001**	0.011*
	Control	165,174 (58772)			
MRT- Mirror Acc (%)	MCI	68.5 (6.51)	221	< 0.001**	0.011*
	Control	79.2 (7.77)			
MRT- Non- Mirror Acc (%)	MCI	89.4 (2.02)	337	0.001*	0.011*
	Control	86.9 (6.81)			
MRT- Mirror RT (msec)	MCI	2861 (337)	138	< 0.001**	0.011*
	Control	1592 (381)			
MRT- Non- Mirror RT (msec)	MCI	1827 (188)	227	< 0.001**	0.011*
	Control	1252 (358)			
Alerting effect (msec)	MCI	0.0242 (0.0489)	155	< 0.001**	0.011*
	Control	-0.0723 (0.0954)			
Orienting Effect (msec)	MCI	0.0656 (0.0232)	137	< 0.001**	0.011*
	Control	-0.0758 (0.102)			
Conflict (msec)	MCI	0.194 (0.0395)	140	< 0.001**	0.011*
	Control	-0.0157 (0.167)			

**p < 0.001, *p < 0.05

RT- Reaction time; Acc- Accuracy; msec- milliseconds

### Exploratory factor analysis

In both groups, factor analysis was conducted using the minimum residual method with oblimin rotation, as it was anticipated that the underlying factors of auditory processing and cognitive ability might be correlated. The analysis excluded variables with factor loadings less than 0.4 [37] to ensure that only those contributing meaningfully to the extracted components are retained.

#### MCI group

EFA yielded a three-factor model with significant results in Bartlett’s test of sphericity (χ² = 1437, df = 325, *p* < 0.001). The Kaiser‒Meyer‒Olkin (KMO) measure of sampling adequacy indicated overall good sampling adequacy (KMO = 0.653), suggesting that the data were suitable for factor analysis. The analysis excluded variables with factor loadings less than 0.4 (QuickSIN-K). The analysis revealed three main factors, which accounted for a cumulative variance of 70.1%. The interfactor correlations revealed poor correlations between the factors, suggesting that there was no overlap between these constructs (refer to supplementary material).

- Factor 1 included temporal processing tests (MDT, FMDL, ATTR, TFS-AF) and cognitive tests corresponding to working memory (DSF RT & length and DSB RT & length), executive function (TMT RT- number and mixed), and visuospatial orientation (MRT- Mirror RT & Acc, and MRT- non mirror RT).
- Factor 2 included dichotic digit scores (DCS, total R, total L, forced RCS and forced LCS) and cognitive tests, including MRT non mirror accuracy and alerting, orienting and conflict effects from the ANT.
- Factor 3 comprised the hearing thresholds (Table 5).

**Table 5:** MCI Factor Loadings

	Factor			
	1	2	3	Uniqueness
Speech PTA R	-0.0167	-0.0735	**0.9460**	0.0773
Speech PTA L	0.0301	0.0330	**0.9258**	0.1590
PTA R	-0.0329	-0.0913	**0.9437**	0.0687
PTA L	0.0287	0.0471	**0.9211**	0.1693
DCS	0.1294	**0.8894**	-0.2034	0.1318
Total R	0.1323	**0.6706**	0.2109	0.5577
Total L	0.0273	**0.8029**	-0.2242	0.2666
Forced RCS	-0.1654	**0.5131**	0.3052	0.6081
Forced LCS	-0.1214	**0.7594**	0.1326	0.3814
FMDL	**0.7821**	0.2560	0.1231	0.4105
ATTR	**0.8506**	0.1184	0.0913	0.3162
MDT	**0.8891**	0.0576	0.0190	0.2275
TFS-AF	**-0.6056**	0.3209	0.0244	0.4695
DSF RT	**0.9675**	-0.0537	-0.0322	0.0332
DSF length	**-0.8537**	0.1909	0.0554	0.1685
DSB RT	**0.9888**	0.0931	-0.0160	0.0333
DSB Length	**-0.9358**	0.0492	0.0421	0.0917
TMT RT number	**0.9921**	0.1406	0.0028	0.0375
TMT RT Mixed	**0.9533**	-0.1217	-0.0229	0.0343
MRT mirror RT	**0.9251**	-0.1602	0.0172	0.0811
MRT mirror Acc	**-0.7105**	-0.1855	0.0247	0.4903
MRT Non-mirror RT	**0.7228**	-0.1227	-0.0685	0.4147
MRT Non-mirror Acc	0.0219	**-0.4092**	0.1339	0.8000
Alerting	0.1531	**-0.4966**	0.0270	0.7058
Orienting	0.3455	**-0.6395**	-0.0342	0.4071
Conflict	0.1170	**-0.5694**	0.0250	0.6401

Note. ‘Minimum residual’ extraction method was used in combination with a ‘oblimin’ rotation

**Abbreviations**: PTA-Pure-tone average, DCS -Double Correct Score in DDT, FMDL- Frequency Modulation Detection Limen, ATTR- Adaptive Temporal Resolution Threshold, MDT- Modulation Detection Threshold, TFS-AF- Temporal Fine Structure Adaptive Frequency, DSF- Digit Span Forward, DSB- Digit Span Backward, MRT- Mental Rotation Task, RT- Reaction Time, Acc - Accuracy

#### Control group

For the control group, EFA yielded a four-factor model with significant results in Bartlett’s test of sphericity (χ² = 1486, df = 300, *p* < 0.001). KMO indicated a good sampling adequacy of 0.618 overall, suggesting that the data were suitable for factor analysis. Variables with factor loadings less than 0.4 (MRT mirror and non mirror Acc) were excluded from the analysis. The analysis identified four main factors, accounting for a cumulative variance of 83%. Factor 1 and Factor 2 had moderate negative correlations (-0.375), whereas the other interfactor correlations were weak (refer to supplementary material).

- Factor 1 included two temporal processing tests (MDT & TFS-AF) and cognitive tests related to working memory (DSF RT & length and DSB RT & length), executive function (TMT RT- number and mixed), and visuospatial orientation (MRT mirror RT & MRT non mirror RT).
- Factor 2 included dichotic digit scores (DCS, total R, total L. forced RCS and forced LCS) and temporal processing tests (FMDL and ATTR).
- Factor 3 comprised the hearing thresholds.
- Factor 4 included alerting, orienting, and conflict measures from the ANT test along with the QuickSIN-K test (Table 6).

**Table 6:** Control factor loadings

	Factor				
	1	2	3	4	Uniqueness
PTA1 R	-0.0703	-0.1937	**0.9025**	-0.0751	0.1010
PTA1 L	0.0848	0.1962	**0.9635**	0.0554	0.0873
Sp PTA R	-0.0680	-0.3443	**0.8520**	-0.0484	0.0840
Sp PTA L	0.1001	0.1270	**0.9654**	0.0515	0.0738
DCS	-0.0215	**0.7060**	-0.1404	0.0163	0.4385
Total R	0.0155	**0.8282**	-0.1702	0.1957	0.2378
Total L	-0.0208	**0.8663**	0.0472	-0.1163	0.2139
Forced RCS	-0.0104	**0.8519**	-0.0425	-0.1168	0.2224
Forced LCS	0.1200	**0.9059**	-0.0104	-0.1425	0.1940
FMDL	0.2806	**-0.7701**	-0.0722	-0.0534	0.1855
ATTR	0.3076	**-0.7417**	-0.0753	-0.0809	0.2012
MDT	**0.7292**	-0.4393	-0.0504	0.0720	0.0525
TFS - AF	**-0.9444**	-0.0509	-0.0341	-0.1599	0.1529
DS-forward RT	**0.9994**	0.0574	0.0134	-0.0326	0.0266
DS-forward Length	**-0.9129**	0.0057	-0.1136	0.0188	0.1169
DS-Backward RT	**0.9446**	-0.0790	-0.0546	-0.0410	0.0454
DS-backward length	**-0.8649**	-0.0495	-0.0475	0.1221	0.2219
TMT RT- number	**0.9873**	0.0273	0.0510	-0.0293	0.0198
TMT RT- mixed	**0.8734**	-0.0734	-4.74e − 4	-0.0942	0.1523
MRT- Mirror RT	**0.9778**	-0.0177	-0.0361	-3.46e − 4	0.0386
MRT- Non-Mirror RT	**0.6727**	0.0291	-0.0765	0.1402	0.5747
QuickSIN-K	0.1298	-0.3052	-0.0405	**0.4030**	0.6907
Alerting	0.0064	0.0029	0.0478	**0.9699**	0.0666
Orienting	-0.0539	-0.0154	-0.0342	**0.9773**	0.0174
Conflict	0.0098	-0.0304	0.0111	**0.9872**	0.0227

Note. ‘Minimum residual’ extraction method was used in combination with a ‘oblimin’ rotation

**Abbreviations**: PTA-Pure-tone average, DCS -Double Correct Score in DDT, FMDL- Frequency Modulation Detection Limen, ATTR- Adaptive Temporal Resolution Threshold, MDT- Modulation Detection Threshold, TFS-AF- Temporal Fine Structure Adaptive Frequency, DSF- Digit Span Forward, DSB- Digit Span Backward, MRT- Mental Rotation Task, RT- Reaction Time, Acc - Accuracy

### Multiple regression analyses

Four multiple regression models were evaluated to determine the predictors of cognition (MoCA scores) in the MCI group. Model 1 included dichotic measures (DCS and REA), Model 2 included QuickSIN-k, Model 3 included temporal processing measures (FMDL, MDT, ATTR, and TFS-AF) and Model 4 included covariates like gender, education and bilateral pure tone threshold average. Model 3 explained the highest percentage of variance (80.7%) [F _(4,30)_ = 14, *p* < 0.001]. Among the temporal processing measures in Model 3, FMDL (β = -0.382, *p* = 0.046) and ATTR (β = -0.529, *p* = 0.012) are significant predictors of cognitive performance (Table 7).

**Table 7:** Model fit measures

**Model 1: Dichotic measures**										
**Model**	***R***	***R*** ^2^		**Overall Model Test**						
				**F**		**df1**		**df2**		***p***
1	0.188	0.035		0.589		2		32		0.561
**Model Coefficients - MoCA**										
**Predictor**	**Estimate**		**SE**		**β**		**t**		**p**	
Intercept	23.2798		0.932		0.000		24.981		< 0.001	
DCS	-0.0980		0.109		-0.190		-0.898		0.376	
REA	-0.1107		0.109		-0.214		-1.013		0.319	

**Model 2: QuickSIN-K**										
**Model**	***R***	***R*** ^**2**^		**Overall Model Test**						
				**F**		**df1**		**df2**		***p***
2	0.158	0.0251		0.850		1		33		0.363
**Model Coefficients - MoCA**										
**Predictor**	**Estimate**		**SE**		**β**		**t**		**p**	
Intercept	23.657		1.465		0.000		16.143		< 0.001	
QuickSIN-k	-0.209		0.226		-0.158		-0.922		0.363	

**Model 3: Temporal Processing measures**										
**Model**	***R***	***R*** ^**2**^		**Overall Model Test**						
				**F**		**df1**		**df2**		***p***
3	0.807	0.651		14		4		30		< 0.001
**Model Coefficients - MoCA**										
**Predictor**	**Estimate**		**SE**		**β**		**t**		**p**	
Intercept	25.40394		1.61827		0.000		15.698		< 0.001	
**FMDL**	**-0.14222**		**0.06836**		**-0.382**		**-2.081**		**0.046**	
**ATTR**	**-0.15143**		**0.05676**		**-0.529**		**-2.668**		**0.012**	
MDT	0.12350		0.14861		0.184		0.831		0.413	
TFS- AF	0.00311		0.00221		0.190		1.410		0.169	

**Model 4: Covariates**									
**Model**	***R***	***R*** ^**2**^		**Overall Model Test**					
				**F**		**df1**		**df2**	***p***
4	0.233	0.0544		0.594		3		31	0.624
**Model Coefficients - MoCA**									
**Predictor**	**Estimate**		**SE**		**β**		**t**		**p**
Intercept	21.11633		2.6486		0.000		7.9727		< 0.001
Gender	-0.59547		0.6953		-0.150		-0.8565		0.398
Education	0.15456		0.1452		0.187		1.0648		0.295
B/L PTA	0.00350		0.0593		0.010		0.0591		0.953

### Receiver operating curve (ROC) analysis

**Fig. 1:**
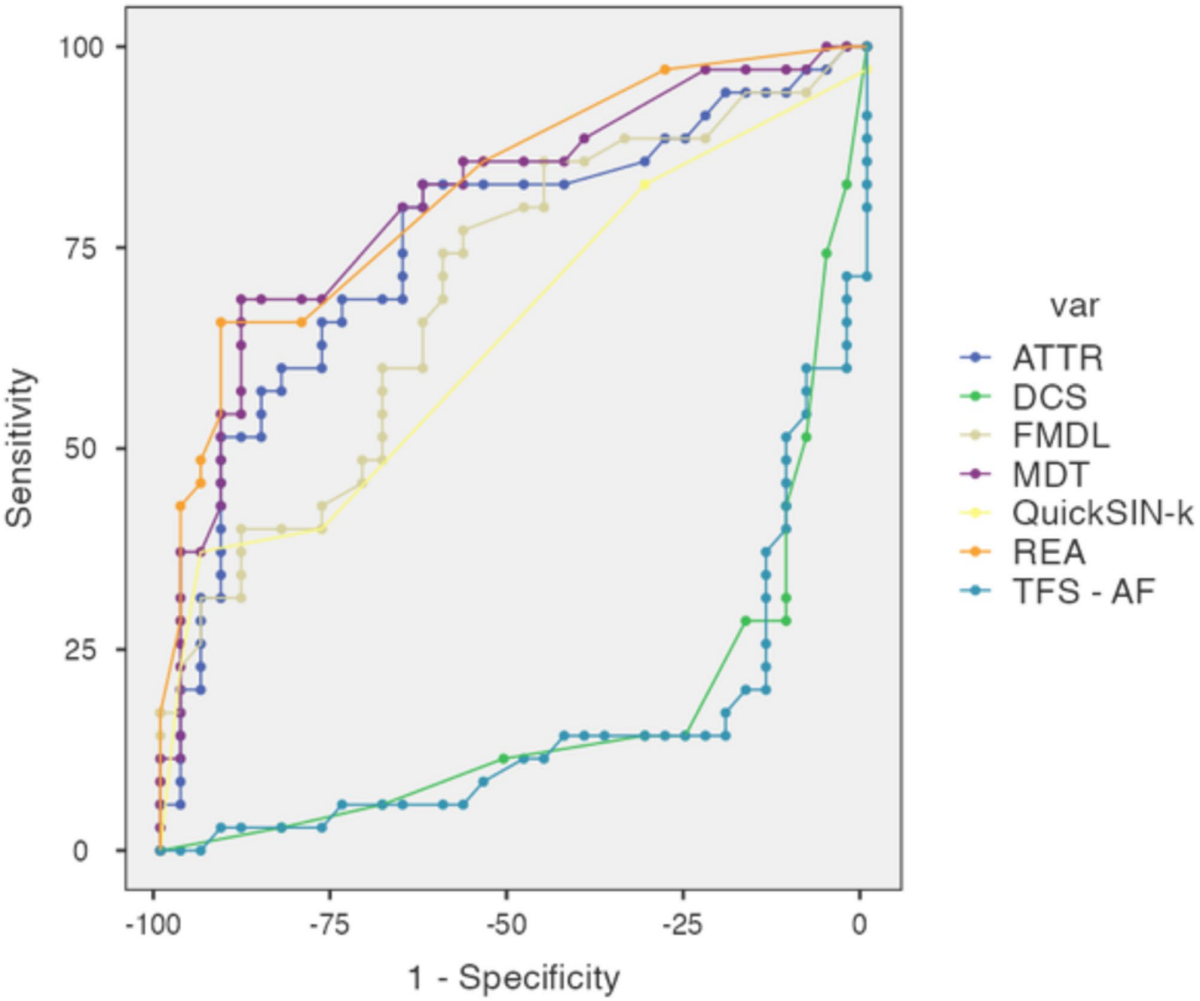
Combined ROC curve for the auditory processing tests

ROC analysis revealed that MDT, REA, FMDL, and ATTR demonstrated good sensitivity and specificity, suggesting excellent discriminability between the two groups. QuickSIN-K showed high specificity (94.29%) but lower sensitivity (37.14%), with an AUC of 0.642, indicating an imbalance (refer to supplementary material). TFS-AF and DCS have very low AUCs of 0.172 and 0.155, respectively, with poor sensitivity and specificity, indicating poor discriminability (refer to Fig. 1).

## Discussion

The present study investigated temporal processing deficits in older adults with MCI compared with cognitively healthy controls. The literature suggests that auditory processing deficits are prevalent among older adults with MCI [21–24, 38]. However, a detailed examination of temporal processing deficits is still lacking. This motivated the current study to assess auditory processing and cognition using a comprehensive battery of tests among older adults with and without MCI.

### Central auditory processing abilities in MCI

To the best of our knowledge, this is the first study to comprehensively assess auditory processing in older adults with MCI. There was a significant deficits across the domains of auditory processing. Individuals with MCI demonstrate significantly poorer performance than controls do in all tests assessing temporal processing. There are very few studies in the literature regarding temporal processing performance in these populations. Existing studies suggest similar deficits when ATTR is used [21]. Temporal processing ability generally decreases with age [2, 39–42]. Our findings indicate that temporal processing deficits in MCI extend beyond typical age-related auditory decline, as evidenced by comparisons with the control group. While normal ageing is associated with declines in central auditory processing, particularly in temporal resolution and frequency modulation detection [12, 43], the MCI group exhibited significantly greater impairments than cognitively healthy older adults.

While we initially observed differences in QuickSIN-k performance between groups, this finding did not remain statistically significant after applying the Holm correction for multiple comparisons (adjusted *p* = 0.064). Nonetheless, the consistent trend of poorer QuickSIN-k scores among older adults with MCI, as seen in both the current study and previous literature [44–46] suggests potential challenges with auditory closure that warrant further investigation with larger sample sizes.

Deficits in DDT were also observed in individuals with MCI, reflecting deficits in binaural integration. These findings are consistent with those of previous studies in the literature [22, 47–50]. The significant deficit in binaural integration may contribute to communication difficulties in challenging listening environments for individuals with MCI. In the dichotic test, a pronounced right ear advantage (REA) was observed in individuals with MCI. This enhanced REA in individuals with neurodegenerative conditions is thought to be explained by a phenomenon called left ear extinction. The size of the corpus callosum decreases with age [51], and this atrophy is more pronounced in individuals with MCI [52]. As a result, stimuli presented to the left ear may not be processed efficiently since they must travel through the corpus callosum for perception. This suggests that enhanced REA in MCI is not due to superior right ear performance but rather the inability to efficiently process left ear inputs.

Previous studies indicate that temporal processing tasks, like frequency modulation detection and gap detection, involve both auditory and cognitive mechanisms [11]. There is no clear distinction between pure auditory processing and cognitive processing, especially in tasks requiring active engagement [53]. However, auditory deficits in MCI may also stem from factors beyond cognitive issues. Electrophysiological studies, such as those measuring MMN, have shown reduced amplitudes and latencies in individuals with MCI, suggesting deficits exist even under passive listening conditions [19]. This implies that while cognitive deficits might lead to variability in behavioural tests, there could also be underlying declines in the neural mechanisms for auditory processing. Future research using objective measures, like EEG assessments during active auditory processing tests, may clarify the influences of cognitive and auditory factors on poor auditory processing performance in MCI.

### Factor structure of CAPD and cognition

Factor analysis, a data-driven approach to assess the underlying construct of APD and cognitive domains, revealed different structures between the two groups. The clustering of attention scores with dichotic scores in older adults with MCI suggests increased reliance on attentional resources to perform complex auditory tasks, such as dichotic listening. This contrasts with the control group, where dichotic scores and attention formed separate, distinct factors, indicating less reliance on attention. These findings support the phenomenon of cognitive dedifferentiation, where an individual’s cognitive abilities become less differentiated as they age, particularly with cognitive decline [54–56]. However, contrary to these findings, a previous study by O’Brien et al. 2020 [53] reported that cognitive domains and auditory processing tests were loaded as separate factors, indicating that they are distinct. One possible reason for the variability is the differences in the methods used for the factor analysis. The current study separately analysed older adults with and without MCI to identify specific population dynamics while considering the subtle variations between the two groups. The very insignificant interfactor correlation suggests nonoverlapping constructs among the factors in both groups. This finding highlights the importance of assessing multiple domains of auditory processing in older adults with and without MCI.

### Significant predictors of cognitive performance

In developmental disorders, CAPD typically affects one or more specific auditory domains rather than impairment across all domains. Therefore, the American Speech and Hearing Association (ASHA) recommends a battery of tests to assess CAPD under those conditions. Although previous studies have explored limited tests of CAPD, findings indicate that older adults with MCI exhibit deficits across all the domains assessed [21–24]. However, the relationships between individual auditory processing domains and cognition in this population are still unclear. To answer this question, four models were tested in a hierarchical model: one with dichotic listening measures, the second with low redundancy (QuickSIN-K), the third with temporal processing and the fourth with covariates. Temporal processing emerged as a significant predictor of cognition. Among the temporal processing measures, only FMDL and ATTR were highly significant. Dichotic measures and the QuickSIN-k score were not significant predictors, although they were significantly different between the two groups.

The prominence of temporal processing impairment aligns with the existing literature that the temporal lobe is particularly vulnerable to early neurodegenerative changes. Neurobiological models suggest that changes in neural synchronization and timing mechanisms can reflect broader cognitive network disruptions [57]. This is particularly relevant in MCI, where early-stage neuronal desynchronization may compromise cognitive function before significant structural brain changes occur [58, 59]. Temporal processing, which involves the brain’s ability to sequence and time auditory information, relies heavily on neuronal precision and synchronization [60, 61]. Thus, deficits in this area could be more directly linked to neuronal disruption seen in MCI.

The selective impairment of temporal processing contrasts with other auditory cognitive tasks, offering insights into the differential vulnerability of neural networks. Dichotic listening and speech-in-noise tasks rely more on complex cognitive control mechanisms such as attention [62] and linguistic processing [63] respectively. While these are affected in MCI, they may not directly correlate with neural desynchronization. Although neural desynchronization can influence these tasks, the relationship is less direct due to the multiple cognitive domains involved. This distinction is crucial, as it suggests that basic neural timing mechanisms may be more sensitive indicators of early cognitive changes than higher-order cognitive functions. However, neurophysiological or imaging studies are needed to confirm the interpretation of neural desynchronization and its relation to temporal processing.

Regardless, it is important to acknowledge certain methodological limitations that may have influenced these results. DDT and QuickSIN tests utilised in this study were translated and validated in Kannada [33, 64]. Despite their clinical sensitivity in other populations, robust psychometric testing specific to older adults is lacking. Further research is needed to address these limitations.

### Clinical sensitivity

Although only a subset of CAPD tests exhibited a linear relationship with cognition, the current study evaluated all the measures for their clinical discriminability between the two groups. Consistent with previous analyses, FMDL and ATTR had good sensitivity and specificity in discriminating older adults with and without MCI. Compared with all other measures, the REA demonstrated superior sensitivity and specificity. This finding suggests that REA effectively distinguishes between two groups; however, it does not demonstrate a linear relationship with cognition. The difference may be attributed to different statistical approaches that assess various aspects of the relationships between variables. Additionally, the mechanisms underlying dichotic listening are complex and require interhemispheric transfer and lateralization [65]. The performance could be influenced by corpus callosum degeneration rather than cognitive impairment [52]. Furthermore, the ceiling effect is commonly reported in dichotic tests, especially the digit version [64, 66, 67]. The results of the present study also revealed that while the control group performed close to the ceiling, the MCI group experienced a significant decrease in scores. This may explain why the test effectively differentiates between the two groups, but it does not indicate a linear relationship with cognitive ability. Therefore, despite its limitations in predicting the extent of cognitive function, the dichotic test, especially the right ear advantage (REA), could still serve as a valuable clinical indicator.

### Strengths and limitations

The key strengths of the current study are the inclusion of comprehensive auditory processing measures and a robust statistical analysis approach. This study has several limitations. First motor speed was not explicitly controlled for, which could have influenced performance on reaction-time-based cognitive tasks. Given that motor function declines with age, future studies should include an independent measure of motor speed (e.g., simple reaction time tasks) to better isolate cognitive processing from motor-related variability. Second, the study did not consider auditory processing, which is governed primarily by lower central auditory structures, such as binaural masking differences. Third, the researchers excluded older adults with hearing impairments to avoid potential confounding variables. As a result, the generalizability of the findings in clinical settings may be limited since most older individuals often exhibit symptoms of peripheral hearing loss. Further research is needed to explore the relationship between auditory processing deficits and cognition in older adults with varying degrees of hearing impairment. The connection between cognition and auditory processing can be understood from both top-down and bottom-up perspectives. Future studies should investigate the directionality of this correlation, as suggested by the cascade hypothesis.

## Conclusion

Overall, the current study demonstrated significant temporal processing deficits in older adults with MCI beyond what is expected for typical age-related decline. The increased REA observed in older adults with MCI could result from left ear extinction. The underlying constructs of auditory processing and cognition differ between older adults with and without MCI. Temporal processing ability demonstrated a significant linear relationship with cognitive performance.

## Electronic supplementary material

Below is the link to the electronic supplementary material.

Supplementary Material 1

Supplementary Material 2

## Data Availability

The datasets used and/or analyzed during the current study are available from the corresponding authors upon reasonable request.

## Abbreviations

CAPD: Central Auditory Processing Disorder
APD: Auditory Processing Disorder
MCI: Mild Cognitive Impairment
SSI-ICM: Synthetic Sentence Identification-Ipsilateral Competing Messaging
SPIN: Speech Perception In Noise
DDT: Dichotic Digit Tests
DCS: Dichotic Correct Scores
REA: Right Ear Advantage
DSI: Dichotic Sentence Identification Test
MoCA: Montreal Cognitive Assessment
PHQ-9: Patient Health Questionnaire-9
QuickSIN-K: Quick Speech-In-Noise Test In Kannada
TFS‒AF: Temporal Fine Structure‒Adaptive Frequency
FMDL: Frequency Modulation Difference Test
ATTR: Adaptive Threshold Of Temporal Resolution
MDT: Modulation Detection Threshold
ANT: Attentional Network Test
MRT: Mental Rotation Test
DSF: Digit Span Forward
DSB: Digit Span Backward
TMT: Trail Making Test
PTA: Pure Tone Average
