# Supplementary material for: Auditory processing and its cognitive correlates in older adults with mild cognitive impairment
Source: BMC Geriatr. 2025 May 24;25:373. doi: 10.1186/s12877-025-05997-4 (PMC12103056; doi:10.1186/s12877-025-05997-4)
Supplement: Supplementary file 1 — Supplementary Material 1 [file 12877_2025_5997_MOESM1_ESM.docx]

DICHOTIC DIGIT TEST SCORES:


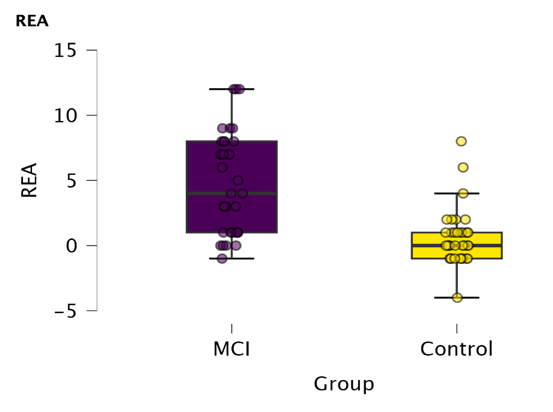

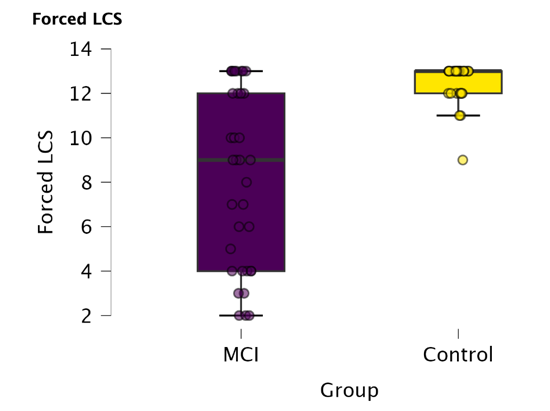

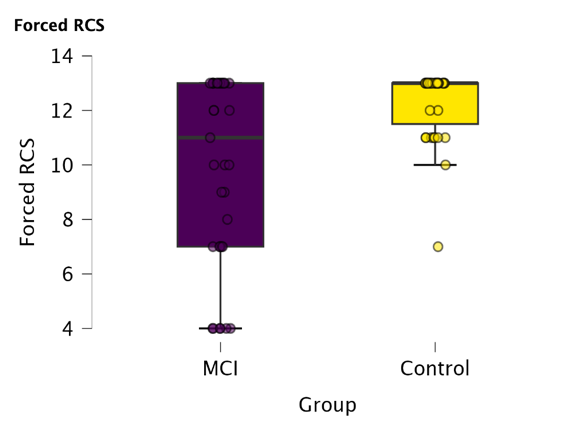

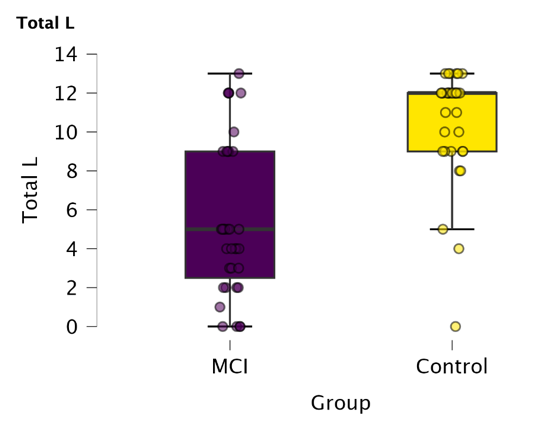

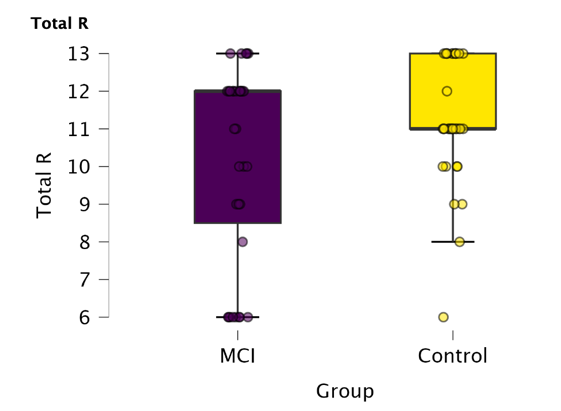

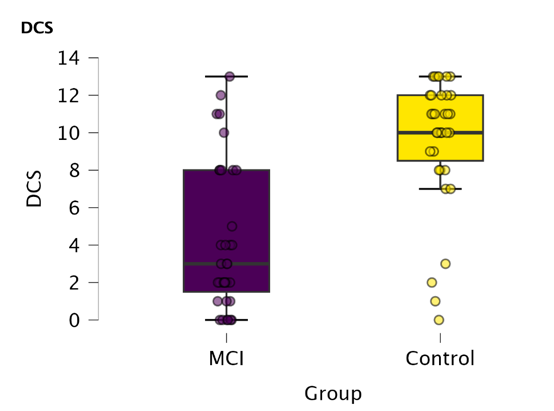


**QUICKSIN:**


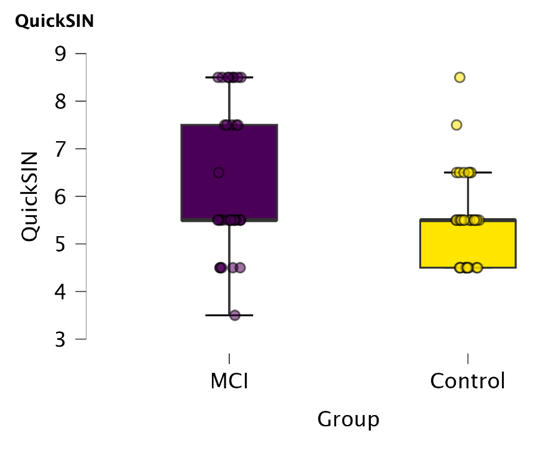


**TEMPORAL PROCESSING MEASURES:**


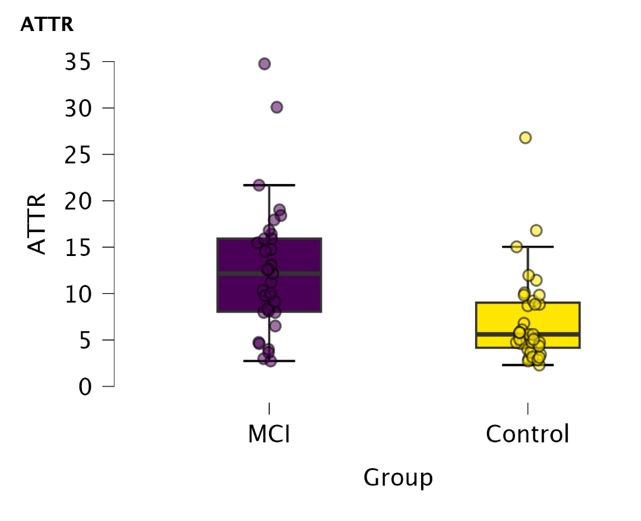

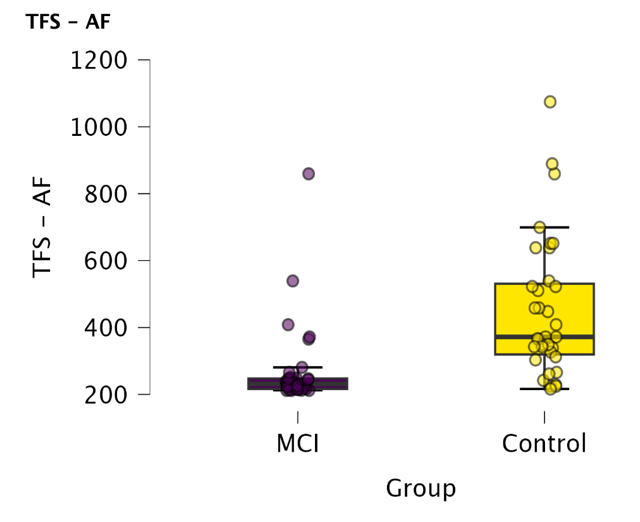


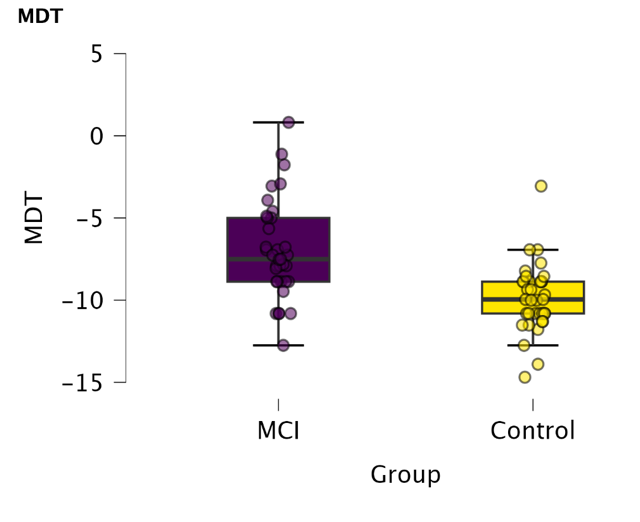

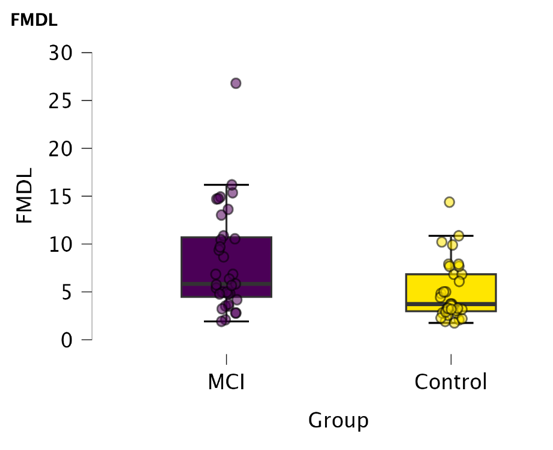


**Cognitive Tests:**

**Digit Span Forward and Backward:**


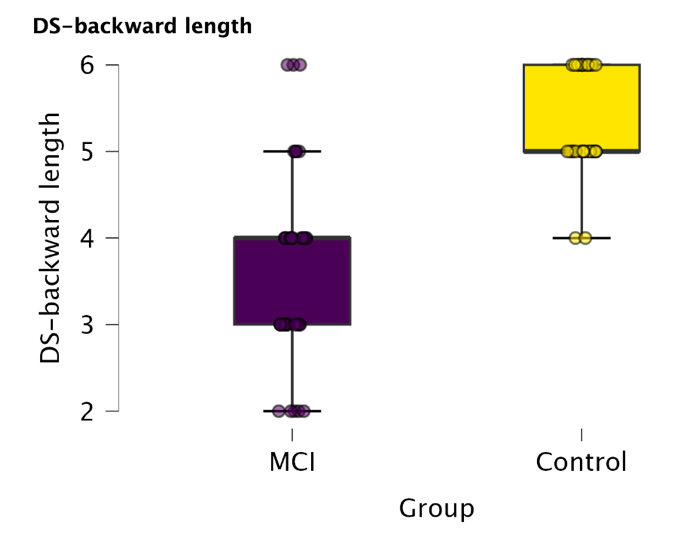


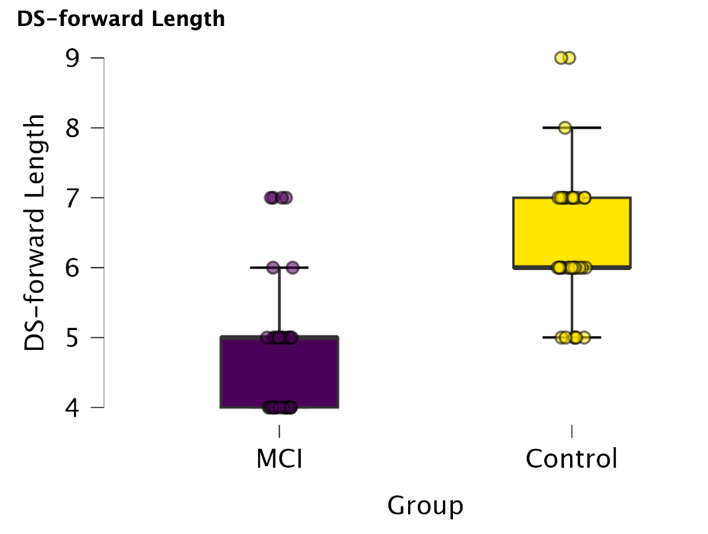


**Trail Making Test A (number) and B (mixed):**


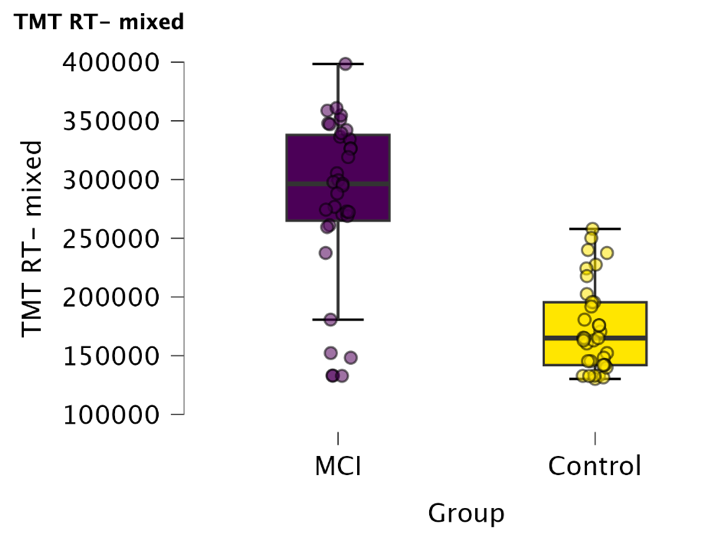

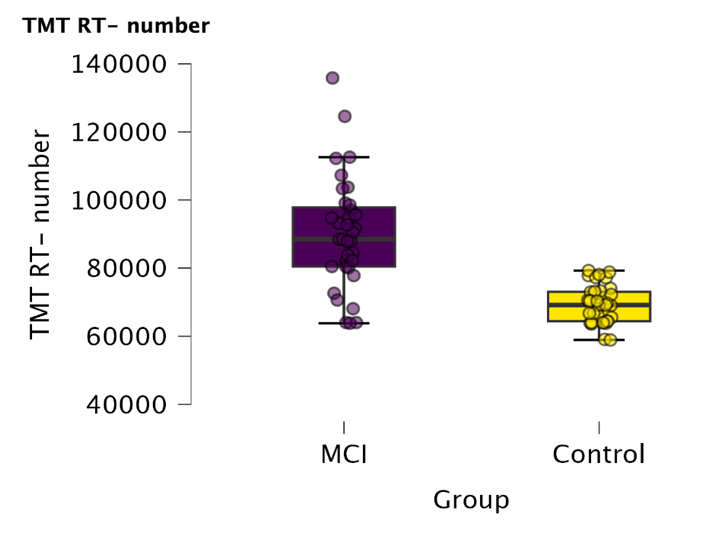


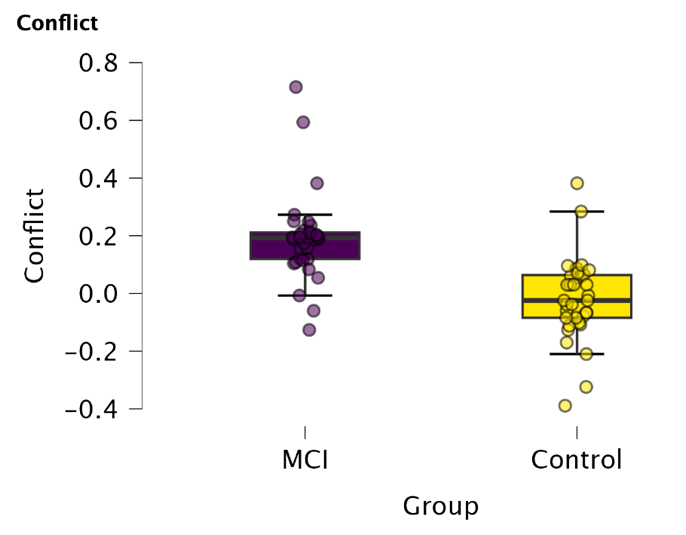

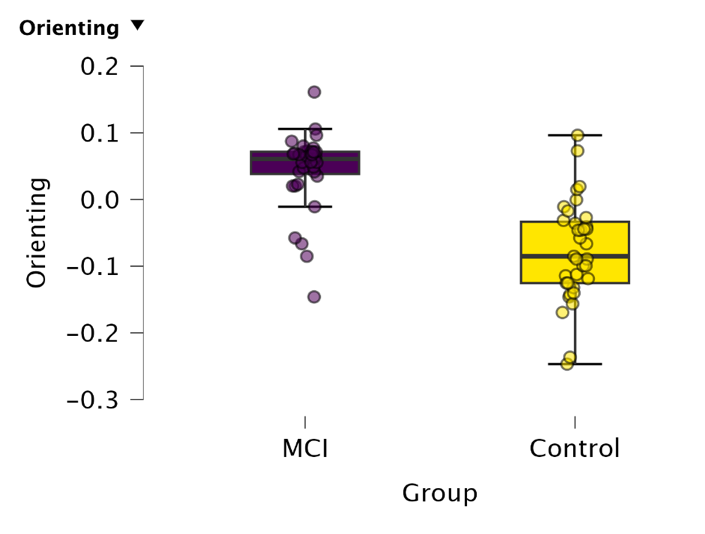

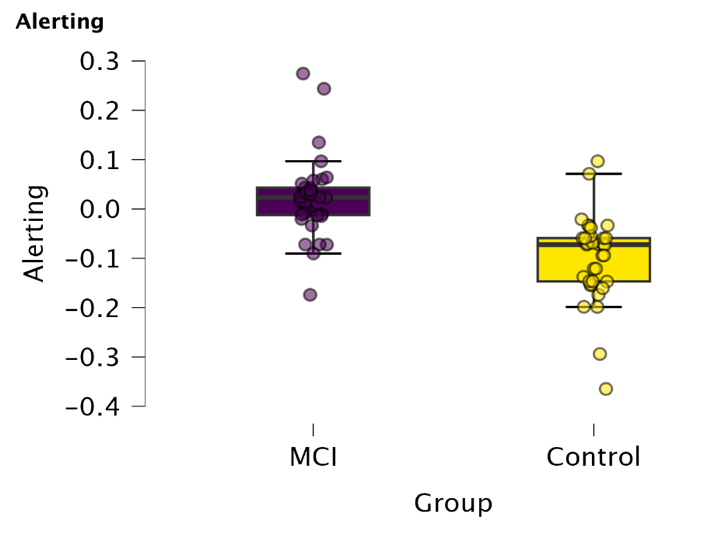
A**ttention Network Test:**
