# Supplementary material for: Auditory processing and its cognitive correlates in older adults with mild cognitive impairment
Source: BMC Geriatr. 2025 May 24;25:373. doi: 10.1186/s12877-025-05997-4 (PMC12103056; doi:10.1186/s12877-025-05997-4)
Supplement: Supplementary file 2 — Supplementary Material 2 [file 12877_2025_5997_MOESM2_ESM.docx]

1. ***Block diagram of ANT paradigm***

**
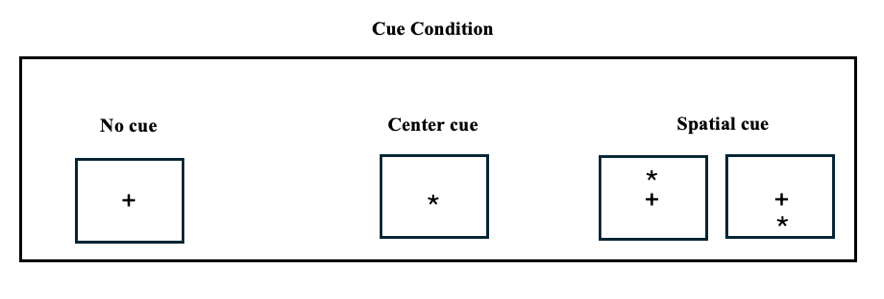
**

**Sequence of events in single trail of ANT**


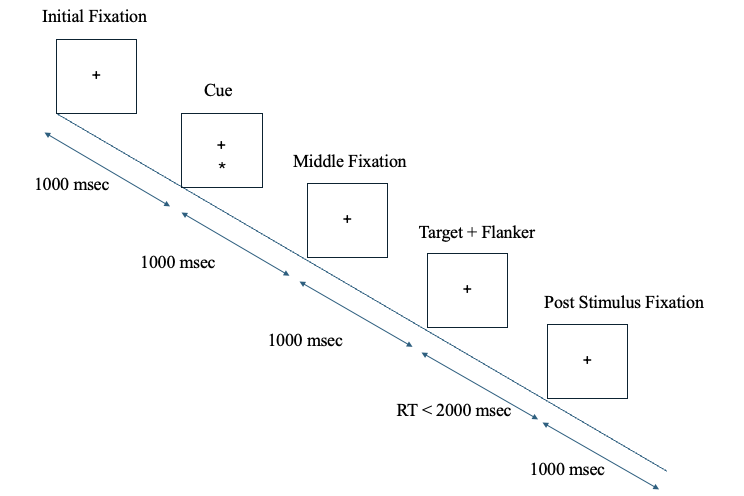


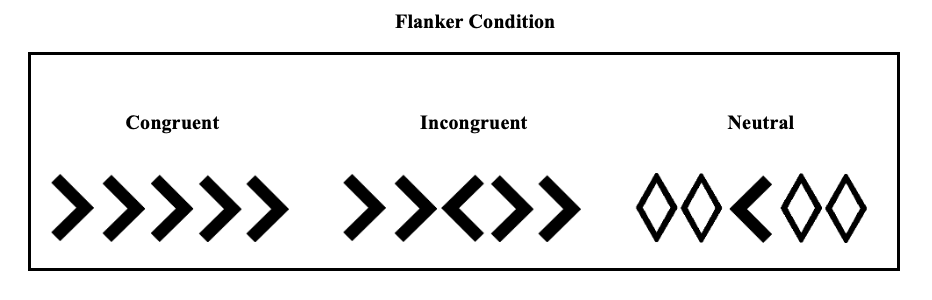


1. ***MCI group- Factor Summary***

| **Factor** | **SS Loadings** | **% of Variance** | **Cumulative %** |
| --- | --- | --- | --- |
| 1 | 10.10 | 38.9 | 38.9 |
| 2 | 4.30 | 16.5 | 55.4 |
| 3 | 3.82 | 14.7 | 70.1 |

**MCI- Inter- factor correlation**

|  | **1** | **2** | **3** |
| --- | --- | --- | --- |
| **1** | - | -0.144 | -0.196 |
| **2** |  | - | -0.116 |
| **3** |  |  | - |

1. **Control group- Factor Summary**

| **Factor** | **SS Loadings** | **% of Variance** | **Cumulative %** |
| --- | --- | --- | --- |
| 1 | 8.53 | 34.1 | 34.1 |
| 2 | 5.44 | 21.7 | 55.8 |
| 3 | 3.55 | 14.2 | 70.1 |
| 4 | 3.24 | 13.0 | 83.0 |

**Control- Inter- factor correlation**

|  | **1** | **2** | **3** | **4** |
| --- | --- | --- | --- | --- |
| **1** | — | -0.375 | 0.130 | -0.1445 |
| **2** |  | — | -0.162 | -0.0953 |
| **3** |  |  | — | -0.0799 |
|  |  |  |  | — |

1. **Receiver Operating Curve analysis:**

| **Scale: DCS** | | | | | | | | | | | | | | | |
| --- | --- | --- | --- | --- | --- | --- | --- | --- | --- | --- | --- | --- | --- | --- | --- |
| **Cutpoint** | | **Sensitivity (%)** | | **Specificity (%)** | | **PPV (%)** | | **NPV (%)** | | **Youden's index** | | **AUC** | | **Metric Score** | |
| 0 |  | 100% |  | 0% |  | 50% |  | NaN% |  | 0.00 |  | 0.172 |  | 1.00 |  |
| Inf |  | 0% |  | 100% |  | NaN% |  | 50% |  | 0.00 |  | 0.172 |  | 1.00 |  |
|  | | | | | | | | | | | | | | | |

| **Scale: REA** | | | | | | | | | | | | | | | |
| --- | --- | --- | --- | --- | --- | --- | --- | --- | --- | --- | --- | --- | --- | --- | --- |
| **Cutpoint** | | **Sensitivity (%)** | | **Specificity (%)** | | **PPV (%)** | | **NPV (%)** | | **Youden's index** | | **AUC** | | **Metric Score** | |
| 3 |  | 65.71% |  | 91.43% |  | 88.46% |  | 72.73% |  | 0.571 |  | 0.821 |  | 1.57 |  |
|  | | | | | | | | | | | | | | | |

| **Scale: QuickSIN-k** | | | | | | | | | | | | | | | |
| --- | --- | --- | --- | --- | --- | --- | --- | --- | --- | --- | --- | --- | --- | --- | --- |
| **Cutpoint** | | **Sensitivity (%)** | | **Specificity (%)** | | **PPV (%)** | | **NPV (%)** | | **Youden's index** | | **AUC** | | **Metric Score** | |
| 7.5 |  | 37.14% |  | 94.29% |  | 86.67% |  | 60% |  | 0.314 |  | 0.642 |  | 1.31 |  |
|  | | | | | | | | | | | | | | | |

| **Scale: FMDL** | | | | | | | | | | | | | | | | | | | | | | | |  |
| --- | --- | --- | --- | --- | --- | --- | --- | --- | --- | --- | --- | --- | --- | --- | --- | --- | --- | --- | --- | --- | --- | --- | --- | --- |
| **Cutpoint** | **Sensitivity (%)** | | | | **Specificity (%)** | | | **PPV (%)** | | | **NPV (%)** | | | **Youden's index** | | | **AUC** | | | **Metric Score** | | |  |  |
| 3.5208 | |  | 85.71% |  | | 45.71% |  | | 61.22% |  | | 76.19% |  | | 0.314 |  | | 0.697 |  | | 1.31 |  | | |
| 4.17915 | |  | 77.14% |  | | 57.14% |  | | 64.29% |  | | 71.43% |  | | 0.343 |  | | 0.697 |  | | 1.34 |  | | |
| 4.436016 | |  | 74.29% |  | | 57.14% |  | | 63.41% |  | | 68.97% |  | | 0.314 |  | | 0.697 |  | | 1.31 |  | | |
| 4.77976 | |  | 74.29% |  | | 60% |  | | 65% |  | | 70% |  | | 0.343 |  | | 0.697 |  | | 1.34 |  | | |
| 4.7942 | |  | 71.43% |  | | 60% |  | | 64.1% |  | | 67.74% |  | | 0.314 |  | | 0.697 |  | | 1.31 |  | | |
|  | | | | | | | | | | | | | | | | | | | | | | | |  |

| **Scale: ATTR** | | | | | | | | | | | | | | | |
| --- | --- | --- | --- | --- | --- | --- | --- | --- | --- | --- | --- | --- | --- | --- | --- |
| **Cutpoint** | | **Sensitivity (%)** | | **Specificity (%)** | | **PPV (%)** | | **NPV (%)** | | **Youden's index** | | **AUC** | | **Metric Score** | |
| 6.095127 |  | 82.86% |  | 60% |  | 67.44% |  | 77.78% |  | 0.429 |  | 0.754 |  | 1.43 |  |
| 6.5085 |  | 82.86% |  | 62.86% |  | 69.05% |  | 78.57% |  | 0.457 |  | 0.754 |  | 1.46 |  |
| 6.797 |  | 80% |  | 62.86% |  | 68.29% |  | 75.86% |  | 0.429 |  | 0.754 |  | 1.43 |  |
| 7.9668 |  | 80% |  | 65.71% |  | 70% |  | 76.67% |  | 0.457 |  | 0.754 |  | 1.46 |  |
| 9.15105 |  | 68.57% |  | 74.29% |  | 72.73% |  | 70.27% |  | 0.429 |  | 0.754 |  | 1.43 |  |
| 9.751809 |  | 65.71% |  | 77.14% |  | 74.19% |  | 69.23% |  | 0.429 |  | 0.754 |  | 1.43 |  |
| 10.0375 |  | 60% |  | 82.86% |  | 77.78% |  | 67.44% |  | 0.429 |  | 0.754 |  | 1.43 |  |
| 10.3353 |  | 57.14% |  | 85.71% |  | 80% |  | 66.67% |  | 0.429 |  | 0.754 |  | 1.43 |  |
| 12.1548 |  | 51.43% |  | 91.43% |  | 85.71% |  | 65.31% |  | 0.429 |  | 0.754 |  | 1.43 |  |
|  | | | | | | | | | | | | | | | |

| **Scale: MDT** | | | | | | | | | | | | | | | |
| --- | --- | --- | --- | --- | --- | --- | --- | --- | --- | --- | --- | --- | --- | --- | --- |
| **Cutpoint** | | **Sensitivity (%)** | | **Specificity (%)** | | **PPV (%)** | | **NPV (%)** | | **Youden's index** | | **AUC** | | **Metric Score** | |
| -8.2278 |  | 68.57% |  | 85.71% |  | 82.76% |  | 73.17% |  | 0.543 |  | 0.805 |  | 1.54 |  |
| -8.066 |  | 68.57% |  | 88.57% |  | 85.71% |  | 73.81% |  | 0.571 |  | 0.805 |  | 1.57 |  |
| -7.909 |  | 65.71% |  | 88.57% |  | 85.19% |  | 72.09% |  | 0.543 |  | 0.805 |  | 1.54 |  |
|  | | | | | | | | | | | | | | | |

| **Scale: TFS - AF** | | | | | | | | | | | | | | | |
| --- | --- | --- | --- | --- | --- | --- | --- | --- | --- | --- | --- | --- | --- | --- | --- |
| **Cutpoint** | | **Sensitivity (%)** | | **Specificity (%)** | | **PPV (%)** | | **NPV (%)** | | **Youden's index** | | **AUC** | | **Metric Score** | |
| 212.132 |  | 100% |  | 0% |  | 50% |  | NaN% |  | 0.0000 |  | 0.155 |  | 1.000 |  |
| 1074.5 |  | 0% |  | 97.14% |  | 0% |  | 49.28% |  | -0.0286 |  | 0.155 |  | 0.971 |  |
| Inf |  | 0% |  | 100% |  | NaN% |  | 50% |  | 0.0000 |  | 0.155 |  | 1.000 |  |
